# Small RNAs in plant defense responses during viral and bacterial interactions: similarities and differences

**DOI:** 10.3389/fpls.2013.00343

**Published:** 2013-09-05

**Authors:** Pablo Peláez, Federico Sanchez

**Affiliations:** Departamento de Biología Molecular de Plantas, Instituto de Biotecnología, Universidad Nacional Autónoma de MéxicoCuernavaca, Morelos, México

**Keywords:** RNA silencing, immunity, susceptibility, plant pathogens, small RNAs

## Abstract

Small non-coding RNAs constitute an important class of gene expression regulators that control different biological processes in most eukaryotes. In plants, several small RNA (sRNA) silencing pathways have evolved to produce a wide range of small RNAs with specialized functions. Evidence for the diverse mode of action of the small RNA pathways has been highlighted during plant–microbe interactions. Host sRNAs and small RNA silencing pathways have been recognized as essential components of plant immunity. One way plants respond and defend against pathogen infections is through the small RNA silencing immune system. To deal with plant defense responses, pathogens have evolved sophisticated mechanisms to avoid and counterattack this defense strategy. The relevance of the small RNA-mediated plant defense responses during viral infections has been well-established. Recent evidence points out its importance also during plant–bacteria interactions. Herein, this review discusses recent findings, similarities and differences about the small RNA-mediated arms race between plants and these two groups of microbes, including the small RNA silencing pathway components that contribute to plant immune responses, the pathogen-responsive endogenous sRNAs and the pathogen-delivered effector proteins.

## INTRODUCTION

Immune responses against pathogens are present in most multicellular organisms. Plants and animals have evolved complex and effective innate immune systems to protect themselves from invading microorganisms (Ausubel, 2005). Plants are capable to fight pathogens through a multilayered innate immune system that accomplishes immune memory and self-tolerance (Spoel and Dong, 2012). The first line of defense against phytopathogens occurs when transmembrane pattern recognition receptors (PRRs) recognize microbial- or pathogen-associated molecular patterns (MAMPs or PAMPs) to trigger a general defense response referred to as PAMP-triggered immunity (PTI). Successful microbes deliver effector proteins into the host cells to suppress PTI resulting in effector-triggered susceptibility (ETS). In turn, many plant species have subsequently evolved a type of immunity triggered by resistance (R) proteins that responds to pathogen effector proteins and overcomes PTI arrest. This type of immunity is called effector-triggered immunity (ETI), and results in disease resistance, usually in consequence of a hypersensitive response (HR) at the infection site. In this co-evolutionary context, natural selection drives pathogens and plants to diversify their effector and resistance genes, respectively (Dangl and Jones, 2001; Jones and Dangl, 2006; Ronald and Beutler, 2010).

Host-encoded small RNAs and small RNA silencing pathway proteins impact factors involved in PTI and ETI (Jin, 2008; Padmanabhan et al., 2009). The small RNA classes work in plant defense responses by causing either post-transcriptional gene silencing (PTGS) or transcriptional gene silencing (TGS) to a set of host or pathogen genes (Baulcombe, 2004). The PTGS process mediated by sRNAs leads to messenger RNA (mRNA) cleavage or translational repression, while the TGS mechanism triggered by sRNAs regulates DNA methylation and histone modifications (Schramke and Allshire, 2004). The small RNA classes that have been identified in plants are: microRNAs (miRNAs), *trans*-acting small interfering RNAs (ta-siRNAs), natural antisense transcript-derived small interfering RNAs (nat-siRNAs), repeat-associated small interfering RNAs (ra-siRNAs) or heterochromatic small interfering RNAs (hc-siRNAs), and long small interfering RNAs (lsiRNAs; Chen, 2009; Vazquez et al., 2010). These regulatory small RNAs are produced through different pathways that share several conserved protein families like: the RNA-dependent RNA polymerases (RDRs), the double-stranded RNA-binding proteins (DRBPs), the Dicer-like proteins (DCLs), the small RNA methyltransferase (HEN1), and the Argonaute proteins (AGOs; Chapman and Carrington, 2007). In general, this group of proteins, together with double-stranded RNA (dsRNA) templates, is able to produce small RNAs with specific sizes to rearrange gene expression and to mount a response during plant–microbe interactions. In this review, the focus is on the differences and the similarities of the plant innate immunity involving host small RNAs, sRNA silencing pathway components, silencing suppressors and microbe-derived sRNAs that have been shown to play a specific role in the interaction of plants with bacteria and viruses.

## SMALL RNA-MEDIATED DEFENSE RESPONSES IN PLANT–MICROBE INTERACTIONS

RNA silencing is an important innate immunity approach used by plants to counteract pathogens including nematodes, fungi, and protists (Katiyar-Agarwal and Jin, 2010). Small RNA-mediated antiviral immunity in plants was observed since the beginnings of RNA silencing molecular characterization (Lindbo et al., 1993; Ratcliff et al., 1997; Wang et al., 2011a). PTGS constitutes a very important line of defense against viruses mainly because these are obligate intracellular pathogens whose life cycle depends on the host cellular functions. Once plant viruses introduce their genetic material of DNA or RNA in either single-stranded or double-stranded form, PTGS and/or TGS are induced to control virus replication and spreading throughout the plant. Similar to endogenous RNA silencing regulation, viral double-stranded RNA triggers the formation of virus-derived small interfering RNAs (vsiRNAs) by DCLs. vsiRNAs are then loaded into AGOs to direct viral DNA or RNA silencing. An important amplification step that promotes production of secondary vsiRNAs and supports the systemic silencing involves the activity of the RDRs. In a certain way, antiviral immunity accomplished through viral RNA silencing could be seen as the exploitation of the host life cycle dependency of viruses. Many biotechnological approaches have taken advantage of this natural occurring antiviral strategy to engineer virus-resistant plants. Interestingly, as usually occurs in plant–viruses interactions, viruses have developed a counter defense strategy based on inhibiting host sRNA-mediated antiviral responses with a diverse class of proteins denominated viral suppressors of RNA silencing (VSRs). An efficient viral suppressor may constitute the difference between an infected or immune plant. Increasing evidence suggests that VSRs may have a strong impact on small RNA silencing pathways and therefore in host endogenous sRNA-regulated processes. In fact, many symptoms of plants during viral diseases are attributed to changes in the regulation of endogenous genes caused by VSRs. Also, some vsiRNAs have been reported to target host genes that may be involved in plant defense responses.

Although RNA silencing seems to be a custom-made defense mechanism against viruses, it also stands out as an important mechanism that bacteria have to overcome in order to cause disease in plants. Host small RNAs have been observed to respond in either viral or bacterial infections to promote plant disease resistance. Many small RNA biogenesis factors have been implicated in regulating plant defense responses to pathogenic bacteria as well. Similar to vsiRNAs, dsRNA from genes of the transfer DNA (T-DNA) of *Agrobacterium tumefaciens* triggers the production of bacteria-derived siRNAs. Similar to viruses, bacteria secrete suppressors of RNA silencing to counteract plants RNA silencing defense responses. Most of the roles of RNA silencing in antibacterial immunity have been described in plant interactions with *Pseudomonas syringae* and *Agrobacterium tumefaciens*. In this section, current research on plant small RNA-mediated immunity against viruses and bacteria, including relevant pathogenic elements triggering plant defense is reviewed. Common similarities and particular differences related to antibacterial and antiviral small RNA-mediated immunity are highlighted.

## HOST SMALL RNAs AND PLANT IMMUNITY AGAINST BACTERIAL AND VIRAL INFECTIONS

Several studies have shown that plant small RNAs are directly involved in bacterial disease responses. The first sRNAs identified to participate in plant immunity during bacterial infections were miRNAs. Plants tested with pathogenic bacteria showed several changes in miRNA accumulation, particularly in auxin signaling related miRNAs (Fahlgren et al., 2007; Jagadeeswaran et al., 2009; Zhang et al., 2011a). Virus-responsive sRNAs have been analyzed during very diverse plant–virus interactions (Kasschau et al., 2003; Chapman et al., 2004; Chen et al., 2004; Dunoyer et al., 2004; Chellappan et al., 2005; Takeda et al., 2005; Zhang et al., 2006; Bazzini et al., 2007, 2009; Bortolamiol et al., 2007; Csorba et al., 2007; Lim et al., 2007; Moissiard et al., 2007; Vogler et al., 2007; He et al., 2008; Azevedo et al., 2010; Naqvi et al., 2010; Perez-Quintero et al., 2010; Varallyay et al., 2010; Amin et al., 2011a,b; Du et al., 2011; Hu et al., 2011; Jay et al., 2011; Lang et al., 2011; Feng et al., 2012; Guo et al., 2012; Li et al., 2012; Pacheco et al., 2012; Shivaprasad et al., 2012; Yifhar et al., 2012). Nevertheless, in contrast to bacterial infections, direct evidence for the specific role of endogenous small RNAs in plant antiviral immunity has been limited due to disturbance of the biogenesis and the function of plant sRNAs by viruses. In several cases, VSRs globally induce or accumulate miRNAs through interaction with components of the miRNA biogenesis pathway that may affect miRNA production, function, or stabilization (Kasschau et al., 2003; Chen et al., 2004; Takeda et al., 2005; Bortolamiol et al., 2007; Csorba et al., 2007; Vogler et al., 2007; Azevedo et al., 2010; Varallyay et al., 2010; Shivaprasad et al., 2012). Similar to a bacterial suppressor that induced transcriptional repression of miR393, viral infections may also alter miRNAs at the transcriptional level (Bazzini et al., 2009). In addition, other viral proteins or satellite RNAs, not necessarily VSRs, may disturb miRNA accumulation (Bazzini et al., 2007; Feng et al., 2012). Several of these disturbances in sRNA accumulation have been correlated with plant symptoms of viral diseases, particularly for alterations of miRNA-target interactions involving the miR156, miR171, miR167, miR390, and miR173 families (Kasschau et al., 2003; Dunoyer et al., 2004; Moissiard et al., 2007; Jay et al., 2011). As a consequence of changes in miRNA accumulation and disturbance of RDR6 or DCL4 by VSRs, changes in accumulation of some miRNA-derived tasiRNAs have been also correlated with phenotypic changes during viral infections (Moissiard et al., 2007; Wang et al., 2010; Yifhar et al., 2012). Furthermore, viral infections can modify hc-siRNAs production and alter the RNA-directed DNA methylation (RdDM) pathway leading to reactivation of transposons and transcription of silenced genes that could negatively impact plant defense responses (Raja et al., 2008; Azevedo et al., 2010; Dowen et al., 2012). Despite the fact that viruses cause broad effects on hosts small RNAs, some specific miRNA families seem to be directly involved in antiviral immunity. Interestingly, several host small RNAs are involved in antibacterial or in antiviral immunity because they regulate R or pathogenesis-related (PR) genes (**Table 1**).

**Table 1:** Plant small RNAs involved in plant immunity.

Small RNA	Target(s)	Host(s)	Pathogen(s)	Reference(s)
miR393	TIR1, AFB2, AFB3, AFB1	*Arabidopsis*	Bacteria	Navarro et al. (2006)
miR160	ARF10, ARF16, ARF17	*Arabidopsis*	Bacteria	Li et al. (2010b)
miR398	CSD1, CSD2, COX5	*Arabidopsis*	Bacteria	Jagadeeswaran et al. (2009); Li et al. (2010b)
miR773	DMT2	*Arabidopsis*	Bacteria	Li et al. (2010b)
miR168	AGO1	*Arabidopsis* and *N. benthamiana*	Viruses	Bortolamiol et al. (2007); Varallyay et al. (2010)
miR162	DCL1	*Arabidopsis*	Viruses	Zhang et al. (2006); Azevedo et al. (2010)
miR482/ miR2118	R genes	*N. benthamiana*	Viruses and Bacteria	Zhai et al. (2011); Shivaprasad et al. (2012)
miR158	PPR gene	*Brassica napus* and *Brassica rapa*	Viruses	He et al. (2008)
miR1885	TIR-NBS-LRR gene	*Brassica napus* and *Brassica rapa*	Viruses	He et al. (2008)
miR393^*^	MEMB12	*Arabidopsis* and *N. benthamiana*	Bacteria	Zhang et al. (2011b)
nta-miR6019	Receptor N	*N. tabacum*	Viruses	Li et al. (2012)
nta-miR6020	Receptor N	*N. tabacum*	Viruses	Li et al. (2012)
nat-siRNAATGB2	PPRL	*Arabidopsis*	Bacteria	Katiyar-Agarwal et al. (2006)
AtlsiRNA-1	AtRAP	*Arabidopsis*	Bacteria	Katiyar-Agarwal et al., 2007)

* Mature star miRNA.

*Host small RNAs with a role in antibacterial and/or antiviral immunity. The plant hosts where the small RNAs came from and/or where functional studies to characterize its roles in plant defenses were performed are specified.*

Perception of the PAMP flagellin by *Arabidopsis* was reported to restrict *P. syringae* invasion; however, no mechanism was known that could be involved in triggering this resistance. Analyzing gene expression profilings of seedlings challenged with flg22, Navarro et al. (2006) observed that the accumulation of three auxin receptor transcripts (*TIR1*, *AFB2*, *AFB3*), targets of miR393, was repressed upon treatment with flg22. However, one of the auxin receptors *AFB1* was not affected possibly due to a slightly different miR393-binding site. This result suggested a role for miR393 in regulating defense responses during *P. syringae* infection. Further experiments confirmed this role for miR393a since its overexpression enhanced plant bacteria resistance and as consequence reduced virulent *P. syringae* pv. tomato (Pst) DC3000 *in planta* growth (Navarro et al., 2006). Besides, expression of mi393a was induced in flg22-stimulated *Arabidopsis* seedlings. When the miR393-resistance auxin receptor *AFB1*-Myc was overexpressed in a *tir1-1* background, it resulted in enhanced disease susceptibility. Interestingly, both virulent Pst DC3000 and avirulent Pst DC3000 carrying the type III effector protein avrRpt2 showed similar growth under the *AFB1*-Myc-overexpressing plants. Accumulation of miR393 was also reported to be induced during infiltration with *Agrobacterium tumefaciens*, the plant pathogen that causes crown gall disease (tumor formation) by transferring bacterial DNA to the plant genome. Both, disarmed and oncogenic strains induced miR393 in an early stage of infection. Interestingly, the flg22 of *Agrobacterium tumefaciens* that is completely inactive to the receptor kinase FLAGELLIN INSENSITIVE2 (*FLS2*) as a ligand, maintained unaltered miR393 expression levels (Pruss et al., 2008). Together, these results suggest that miR393a is clearly involved in ETI and, most importantly, that repression of auxin signaling constitutes a plants defense response to bacterial infection.

Following high-throughput sequencing analyses have supported the upregulation of miR393 during plant–bacteria interactions (Fahlgren et al., 2007; Li et al., 2010b; Zhang et al., 2011b). In addition to miR393, the small RNAs miR160 and miR167, regulators of auxin response factors (ARFs) and also members of the auxin signaling pathway were induced after non-pathogenic Pst DC3000 *hrcC*^-^ inoculation and flg22 treatment. Surprisingly, treatment with flg22 did not show a significant reduction of miR167 targets *ARF8* and *ARF6*, in contrast to the downregulation of miR160 targets *ARF10*, *ARF16*, *ARF17*. Plants overexpressing miR160a that were treated with flg22 and *hrcC*^-^ mutant bacteria increased callose deposition. Nevertheless, in miR160 overexpressing plants, resistance to Pst DC3000 bacteria proliferation was not affected (Li et al., 2010b).

As well as increasing accumulation of hormone-related miRNAs contributes to plant defense, downregulation of certain miRNAs impact plant immunity (Sunkar et al., 2006; Jagadeeswaran et al., 2009; Li et al., 2010b). For instance, miR398, whose targets are two copper superoxide dismutases (*CSD1,CSD2*) and a cytochrome c oxidase subunit V (*COX5*), is reduced only in plants challenged with avirulent strains such as Pst DC3000 *avrRpm1* and Pst DC3000 *avrRpt2* (Jagadeeswaran et al., 2009). Accumulation of miR398 is also changed by abiotic and biotic stresses such as salinity, increased light, increased Cu^2+^ and Fe^3+^, ozone stress, and flg22 treatment (Sunkar et al., 2006; Jagadeeswaran et al., 2009; Li et al., 2010b). During biotic and abiotic stresses plants induce early and rapid accumulation of reactive oxygen species (ROS) in the infection zone. Superoxide dismutases (SOD) enzymes process superoxide into oxygen and hydrogen peroxide and therefore regulate ROS. Expression of miR398 is reduced in oxidative stress, promoting accumulation of *CSD1* and *CSD2* (Sunkar et al., 2006). In accordance with expression analyses in different stresses, overexpression of miR398 reduced callose deposition after flg22 treatment and Pst DC3000 *hrC*^-^ infection. Moreover, these transgenic plants were more susceptible to virulent and avirulent strains of *P. syringae* as consequence of *CSD1*, *CSD2* and *COX5* gene silencing (Li et al., 2010b). These last observations confirmed a link between the miR398 family and miRNA-mediated plant defense responses.

Another reported miRNA that is involved in PTI is miR773 (Li et al., 2010b). This miRNA targets the mRNA coding for the DNA methyltransferase 2 (*DMT2*). RNAi-mediated gene silencing of *DMT2*, and a different DNA methyltransferase (*DMT1*), results in reduced tumor formation during *Agrobacterium* infection (Crane and Gelvin, 2007). Interestingly, *MET1* is downregulated in response to biotic and salicylic acid (SA) stresses (Dowen et al., 2012). Deep sequencing analysis of AGO1-immunoprecipitated small RNAs revealed a reduction in miR773 accumulation after flg22 treatment. In accordance, accumulation of the miRNA target *DMT2* was induced in response to flg22 treatment. Transgenic plants overexpressing miR773 showed reduced *MET2* mRNA levels, reduced callose deposition and enhanced susceptibility to the Pst DC3000 and Pst DC3000 *hrC*^-^ strains. Reduction of miR398 and miR773 upon biotic stress exemplifies a negatively regulation of PTI (Li et al., 2010b).

Maintenance of AGO1 homeostasis in plants depends on the *AGO1*-mediated stabilization of miR168 and on the miR168-mediated cleavage of *AGO1* mRNA guided by the same AGO1 protein (Rhoades et al., 2002). Viral infections in plants induce the accumulation of both the *AGO1* mRNA and the miR168 mature miRNA (Bortolamiol et al., 2007; Varallyay et al., 2010). Induction of *AGO1* mRNA during viral infections is considered a plant defense response, while accumulation of miR168 is a viral counterstrategy. Increasing *AGO1* mRNA is a recurrent plant defense reaction toward viral infections because AGO1 protein guides vsiRNAs against viral RNAs. Interestingly, induction of miR168 was spatially correlated with *Tombusvirus* accumulation carrying the p19 suppressor. The specific role of miR168 in plant–virus interactions was further supported when the viral suppressor p19 was removed from this virus and no accumulation of miR168 was observed. Failure of viruses to induce miR168 accumulation indeed promotes accumulation of AGO1 protein and results in a stronger antiviral response (Varallyay et al., 2010).

The miRNA miR162 is also a miRNA that regulates an important component of the miRNA biogenesis pathway so, it is therefore not surprising that this miRNA constitutes a relevant sRNA in antiviral responses as well (Zhang et al., 2006; Azevedo et al., 2010). The mRNA of *DCL1* is regulated by miR162 loaded into AGO1 (Xie et al., 2003). In plant–virus interactions, several VSRs impaired AGO1 activity enhancing *DCL1* mRNA accumulation (Xie et al., 2003; Zhang et al., 2006; Azevedo et al., 2010). Unexpectedly, the accumulation of *DCL1* in *Arabidopsis* plants infected with the *Turnip crinkle virus *(TCV; carrying the P38 viral suppressor) favors reduction of DCL4 and DCL3 two important DCLs in charge of producing vsiRNAs (Azevedo et al., 2010). Although miR162 accumulation in plants infected with the TCV was reduced, it was determined that transcriptional enhancement of *DCL1* is mainly a consequence of inhibition of AGO1 activity. In this sense, the antiviral role of miR162 is coupled with disturbance of AGO1 activity and involves regulation of DCLs. It would be interesting to determine if miR838, other miRNA that regulates *DCL1*, could also be related to antiviral immunity (Rajagopalan et al., 2006).

Ultimately, a group of miRNA families have been reported in legumes and tomato to be directly involved in ETI by regulating many R genes of the NBS-LRR class (Zhai et al., 2011; Shivaprasad et al., 2012). The NBS-LRR proteins activate plant defense responses through recognition of microbe effectors to initiate usually a race-specific ETI. The conserved miRNA families reported to regulate many of these genes are mainly three: miR1507, miR2109, and the superfamily miR482–miR2118 (due to similar sequence members). Most of the members of these families have been detected in legumes. Several of the R target genes identified in these studies produced phased siRNAs as consequence of the 22-nt size of the miRNA regulator members. In some cases, the phased loci (NBS-LRR genes) producing secondary siRNAs presented the “two-hit” model of two miRNA target sites in the mRNA. Identification of phased R loci in *M. truncatula*, soybean, and tomato showed variability in the number of regulated NBS-LRR genes by these miRNA families between species. Based on the constitutive accumulation of some members of these families in tomato and legumes, it is considered that most of the reported miRNA-regulated NBS-LRR targets are silenced in absence of a pathogenic agent (Zhai et al., 2011; Shivaprasad et al., 2012). In this regard, Shivaprasad et al. (2012) found that miRNA-mediated silencing of two disease resistance mRNAs by the miR482–miR2118 superfamily was decreased in plants infected with *P. syringae* DC3000. Similar to bacterial infections, leaves of tomato inoculated with the TCV, the *Cucumber mosaic virus* (CMV) and the *Tobacco rattle virus* (TRV) presented reduced accumulation of miR482. In accordance, two miR482 NBS-LRR mRNA targets were induced in infected plants, especially in plants infected with TCV. A secondary siRNA, product of one of these phased resistance loci that targets a defense-related mRNA and forms a sRNA regulatory cascade, was also suppressed during viral and bacterial infections. Taking into account these observations, they proposed that miRNA-regulated R genes might participate in an uncommon non-race-specific immunity mechanism when this small RNA regulation is blocked by pathogen-encoded suppressors of RNA silencing to release the defense resistance targets. Although further experiments are necessary to extensively validate this hypothesis, previous observations suggest this could be a possible defense mechanism. For example, Li et al. (2012) established other cases for miRNA regulation of innate immune receptors (NBS-LRR genes) in tobacco. Likewise, they showed that increased miRNA repression of a specific R gene attenuates resistance to the *Tobacco mosaic virus *(TMV) in *Nicotiana benthamiana*. Additionally, it was previously reported that overexpression of miR482 miRNA resulted in hypernodulated soybeans, that miR482 is induced after *Bradyrhizobium japonicum* inoculation, and that miR1507 accumulates upon rhizobia infection in the roots of a supernodulated mutant (Li et al., 2010a).

Other small RNAs that are involved in ETI during virus infections are miR158 and miR1885. Inoculation assays performed in *Brassica napus* and *Brassica rapa* with the *Turnip mosaic virus* (TuMV) revealed an enhanced accumulation of miR158 and miR1885 (He et al., 2008). Surprisingly, these miRNAs are generated from the same precursor. The sRNA miR1885 was predicted to target TIR-NBS-LRR class disease-resistant transcripts in *Brassica*, while miR158 targets mRNAs of pentatricopeptide repeat (PPR) containing proteins in *Arabidopsis*. However, further studies are still needed to determine the precise role of these miRNAs in antiviral immunity besides the identity of their targets because it is well-known that the viral suppressor of TuMV HC-Pro increased miRNA accumulation and inhibits miRNA cleavage function (Kasschau et al., 2003).

In the beginning of miRNAs characterization, it was conceived that only one of the strands of the sRNA duplexes was functional and therefore was selected to be loaded into the RNA-induced silencing complex (RISC) to regulate gene expression. Now, several studies have confirmed that the opposite strand (star or passenger strand) of a defined miRNA could also be functional in plants and animals. In relation with small RNA-mediated plant defense responses, the star strand miR393* has also been shown to play a role in plant immunity (Zhang et al., 2011b). In *Arabidopsis*, AGO2 is strongly induced after *P. syringae* pv. tomato contact. One of the star strands abundantly loaded into AGO2 after Pst *avrRpt2* treatment was miR393*. Among three predicted targets for this miRNA, one target experimentally validated for this miRNA, and predicted to have a function in vesicle transport, is *MEMB12*, a Golgi-localized SNARE protein. The knockout mutant of *memb12* exhibit enhanced resistance to avirulent and virulent strains of *P. syringae*. The plant secretory machinery has been proposed as a critical mechanism in plant–microbe interactions due to its capacity to secrete antimicrobial proteins and several biomolecules. During loss-of-function of MEMB12, it was observed that the major secreted antimicrobial pathogenesis-related protein PR1 was strongly secreted. This result suggests that enhanced resistance conferred by the mutant *memb12* is a consequence of the accumulation and secretion of PR proteins. As expected, overexpression of miR393* also showed enhanced disease resistance to Pst *avrRpt2* and also presented increased accumulation and secretion of the PR1 protein (Zhang et al., 2011b).

Lately, two miRNAs named nta-miR6019 and nta-miR6020 were reported to target transcripts of the TIR-NB-LRR immune receptor *N* in tobacco. The immune receptor *N* was the first virus-related resistance gene identified. It confers resistance to the TMV. Expression of these two miRNAs in *N. Benthamiana* was found to reduce *N*-mediated resistance to the TMV. Interestingly, the 22-nt miRNA nta-miR6019 triggers production of secondary siRNAs from the *N* gene similar to the miR482 miRNA family. In this study, eight more miRNA families from tobacco, tomato, and potato that may target R genes were identified, including the miR482 family (Li et al., 2012).

Just as miRNAs contribute to plant immunity, other host siRNAs also promote gene expression reorganization during plant defense responses. These small RNAs are induced in response to pathogenic bacteria and are implicated in triggering plant disease resistance. As mentioned before, the type of siRNAs that have been described to participate in plant immune responses are the nat-siRNA nat-siRNAATGB2 and the bacteria-induced long siRNAs (mainly AtlsiRNA-1; Katiyar-Agarwal et al., 2006, 2007).

Previous to the discovery of nat-siRNAATGB2, siRNAs were known to be involved in antiviral defense mechanisms in plants and animals. Even so, there was a lack of information regarding siRNA-mediated regulation of antibacterial defenses in plants. In this context, finding of nat-siRNAATGB2 revealed, for the first time, the importance of siRNAs and, particularly nat-siRNAs, in controlling antibacterial immunity in plants (Katiyar-Agarwal et al., 2006). This siRNA is generated from the overlapping region of two NATs: *ATGB2* (Rab2-like small GTP-binding protein gene) and *PPRL* (pentatricopeptide repeat-like gene). It targets the 3′ UTR region of *PPRL*. In plants infected with Pst (*avrRpt2*), nat-siRNAATGB2 strongly and specifically accumulates. Surprisingly, this siRNA has a role in disease resistance against pathogenic bacteria by silencing the *PPRL* gene which negatively regulates the coiled-coil NBS-LRR type R protein RPS2. Induction of this siRNA also depends on the disease resistance gene *RPS2* and on the *NDR1* gene that is required for RPS2-specified resistance. Plants overexpressing a resistant version of *PPRL* (without UTR) against nat-siRNAATGB2 that were inoculated with Pst *avrRpto* showed delayed HR, reduced level of cell death and enhanced pathogen growth. All these results suggest that nat-siRNAATGB2 plays a positive role in disease resistance through regulation of *PPRL*.

In an effort to identify other small RNAs specifically induced in a pathogen interaction (*P. syringae*), Katiyar-Agarwal et al. (2007) discovered a class of small RNAs (lsiRNAs). Of the six lsiRNAs discovered, five are induced in response to Pst (*avrRpt2*) infection. The most functionally characterized lsiRNA is AtlsiRNA-1. This siRNA is generated from the overlapping region of the *SRRLK* (putative leucine-rich repeat receptor-like protein kinase) and *atRAP* (an expressed protein that contains a putative RNA-binding domain) natural antisense transcripts. AtlsiRNA-1 is complementary to the 3′ UTR of the antisense gene *AtRAP* and therefore regulates its expression. In mutant *atrap* plants less growth of both virulent and avirulent Pst was observed, suggesting a negative regulatory role for this gene in plant resistance responses. Based on these results, AtlsiRNA-1 may promote resistance against Pst *avrRpt2* infection because of the particular regulation of its target. Almost all the discovered AtlsiRNAs still have to be confirmed to play a role in plant defense responses. Nonetheless, taking into account the cases exposed here of siRNAs related to plant immunity during bacterial infections, together with the growing knowledge generated around small RNAs, for example Pol IV-dependent siRNAs (p4-siRNAs), more siRNAs could also be involved in regulating bacteria stress responses.

## ANTIVIRAL AND ANTIBACTERIAL ROLES OF THE SMALL RNA BIOGENESIS FACTORS

Previously described sRNAs that play important roles in bacterial and viral infections are produced by diverse small RNA silencing pathways. Some small RNA biogenesis factors are directly involved in plant immunity against pathogens (**Table 2**). Consequently, the sRNA silencing pathway components may be altered during bacterial infection to precisely affect production of the sRNAs that contribute to plant resistance. Besides, many components of the small RNA silencing pathways involved in PTGS form the antiviral RNA silencing defense mechanism. Functional studies have assigned a concise or a redundant function in antiviral immunity to various elements of the sRNA silencing pathways. The importance of some of these elements in regulating antiviral plant defenses is reflected in the attempt of many viral suppressors to disturb their activity. Coincidentally, some of these small RNA biogenesis factors have a dual role in plant defense responses against both bacterial and viral infections.

**Table 2:** Small RNA biogenesis factors involved in plant defense.

Protein	Bacteria	Viruses	Reference(s)
DCL1	✔	✔	Dunoyer et al. (2006); Katiyar-Agarwal et al. (2006, 2007); Navarro et al. (2008); Qu et al. (2008); Azevedo et al. (2010); Li et al. (2010b)
HEN1	✔	✔	Boutet et al. (2003); Blevins et al. (2006); Katiyar-Agarwal et al. (2006, 2007); Navarro et al. (2008); Jamous et al. (2011); Zhang et al. (2012)
RDR6	✔	✔	Mourrain et al. (2000); Muangsan et al. (2004); Qu et al. (2005, 2008); Schwach et al. (2005); Adenot et al. (2006); Dunoyer et al. (2006); Katiyar-Agarwal et al. (2006, 2007); Donaire et al. (2008); Garcia-Ruiz et al. (2010); Wang et al. (2010, 2011b)
AGO1	✔	✔	Morel et al. (2002); Mi et al. (2008); Qu et al. (2008); Azevedo et al. (2010); Li et al. (2010b);Wang et al. (2011b); Garcia et al. (2012)
AGO2	✔	✔	Harvey et al. (2011); Jaubert et al. (2011); Scholthof et al. (2011); Wang et al. (2011b); Zhang et al. (2011b, 2012); Carbonell et al. (2012)
AGO4	✔	✔	Agorio and Vera (2007); Raja et al. (2008); Bhattacharjee et al. (2009); Duan et al. (2012); Hamera et al. (2012)
AGO7	✔	✔	Katiyar-Agarwal et al. (2007); Qu et al. (2008)
Pol V	✔		López et al. (2011)
AGO5		✔	Takeda et al. (2008)
DCL4	✔	✔	Blevins et al. (2006); Bouché et al. (2006); Deleris et al. (2006); Katiyar-Agarwal et al. (2007); Qu et al. (2008); Garcia-Ruiz et al. (2010)
DCL3		✔	Qu et al. (2008); Raja et al. (2008); Azevedo et al. (2010)
DCL2		✔	Xie et al. (2004); Bouché et al. (2006); Deleris et al. (2006); Qu et al. (2008); Garcia-Ruiz et al. (2010); Zhang et al. (2012)
RDR1		✔	Xie et al. (2001); Yu et al. (2003); Donaire et al. (2008); Garcia-Ruiz et al. (2010); He et al. (2010);Wang et al. (2010)
RDR2		✔	Donaire et al. (2008); Garcia-Ruiz et al. (2010)
SDE3		✔	Dalmay et al. (2001); Garcia et al. (2012)
SDE5		✔	Hernandez-Pinzon et al. (2007)
SGS3	✔	✔	Muangsan et al. (2004); Adenot et al. (2006); Katiyar-Agarwal et al. (2006, 2007)
HYL1	✔		Katiyar-Agarwal et al. (2006, 2007)

*Small RNA biogenesis factors involved in plant immunity against viruses and bacteria. Check mark (✔) denote its participation in plant defense responses during viral or bacterial interactions.*

Analyses performed in *Arabidopsis thaliana* of mutants corresponding to the sRNA biogenesis factors have provided different clues about their function in plant immunity. DCL1 and HEN1 have been observed to play important roles in PTI and ETI. Growth of Pst DC3000 *hrcC*^-^, a strain capable to trigger PTI but unable to fight it, was enhanced in *dcl1-9* and *hen1-1* mutants. Also, in these mutants, the *P. syringae* pv. phaseolicola (Psp) strain NPS3121, which does not infect *Arabidopsis*, the non-pathogenic *P. fluorescens* Pf-5 and the *E. coli* W3110 strains showed enhanced growth (Navarro et al., 2008). Besides, *dcl1-9* mutants pretreated with flg22 failed to increase resistance to Pst DC3000 and to induce callose deposition (Li et al., 2010b). The impact of DCL1 and HEN1 in plant immunity also has been tested during *Agrobacterium tumefaciens* infections. Roots and stems of *dcl1 *and *hen1* mutants were immune to infection. This means that no tumor growth was observed and that tumor induction by *Agrobacterium tumefaciens* probably requires miRNA adequate functioning. Conversely, *rdr6* mutants were reported to be more susceptible to *Agrobacterium tumefaciens* (Dunoyer et al., 2006). DCL1, HYPONASTIC LEAVES 1 (HYL1), HEN1, SUPPRESSOR OF GENE SILENCING 3 (SGS3), and RDR6 are involved also in plant defense responses due to their role in the biogenesis of the NAT-siRNA nat-siRNAATGB2 and the bacteria-induced lsiRNA AtlsiRNA-1 (Katiyar-Agarwal et al., 2006, 2007). The accumulation of AtlsiRNA-1 was also reduced in a *dcl4-2* mutant so, DCL4 is considered to function in antibacterial plant responses (Katiyar-Agarwal et al., 2007).

As expected, the AGO proteins, the most important components of the RISC, participate in plant resistance to bacteria as well. AGO1 is required in the seedling growth inhibition process determined by flg22 treatment. Also, as a result of analyzing the expression of two PAMP-response genes, it was proposed that AGO1 is necessary for flg22-induced gene expression. The *ago1-25* and the *ago1-27* mutants, like the *dcl1-9* mutants, showed reduced callose deposition and failed to increase resistance to Pst DC3000 (Li et al., 2010b). Besides, AGO1 contributes to antibacterial responses by loading miR393 into the RISC (Mi et al., 2008). AGO7 also plays an antibacterial function through its participation in the biogenesis of AtlsiRNA-1 (Katiyar-Agarwal et al., 2007). An *ago7* mutant resulted in increased susceptibility to Pst (*avrRpt2*; Li et al., 2010b). The argonaute protein AGO2 provides antibacterial resistance by carrying miR393b* to regulate exocytosis of antimicrobial PR proteins. AGO2 is highly induced by Pst to function in innate immune responses. Furthermore, the *ago2-1* mutant displayed enhanced susceptibility to Pst (*avrRpt2* and *EV*; Zhang et al., 2011b).

Small RNA biogenesis factors that are involved in the biogenesis of hc-siRNAs and in RdDM are also essential for antibacterial resistance. The *ago4-2* mutant in *Arabidopsis thaliana* showed enhanced susceptibility to the pathogenic bacteria Pst DC3000, to the avirulent bacteria Pst DC3000 (*avrRpm1*) and to the non-host pathogen *P. syringae* pv. tabaci (Agorio and Vera, 2007). Interestingly, loss-of-function of other components of the RdDM pathway working upstream and downstream of AGO4 did not affected susceptibility to Pst DC3000. Moreover, pol V mutants showed enhanced disease resistance against *P. syringae *DC3000 and enhanced SA-mediated defense responses (López et al., 2011). These results suggest that the RdDM pathway may regulate antibacterial immune responses in plants.

Argonaute proteins in *Arabidopsis* play important roles in plant resistance to viruses as well (Morel et al., 2002; Zhang et al., 2006, 2012; Pantaleo et al., 2007; Qu et al., 2008; Raja et al., 2008; Takeda et al., 2008; Bhattacharjee et al., 2009; Azevedo et al., 2010; Harvey et al., 2011; Jaubert et al., 2011; Scholthof et al., 2011; Wang et al., 2011b; Carbonell et al., 2012; Duan et al., 2012; Garcia et al., 2012; Hamera et al., 2012). AGO1 is a key component in this response. AGO1 is able to load vsiRNAs and target viral RNAs (Azevedo et al., 2010; Garcia et al., 2012). It is a protein highly targeted by VSRs to inhibit its cleavage activity or promote its degradation (Bortolamiol et al., 2007; Csorba et al., 2007; Azevedo et al., 2010). As expected, susceptibility to viruses was increased in *ago1* mutants (Morel et al., 2002). Also, AGO1 participates in the removal of viral RNAs, mainly those with more compact secondary structures (Qu et al., 2008). In addition, as previously mentioned, AGO1 constitutes a master regulator of a complex network that involves DCLs and AGO2 (regulated by miR403 in an AGO1-dependent manner; Azevedo et al., 2010). Interestingly, AGO1 and AGO2 act together in a non-redundant way against the CMV lacking the 2b suppressor downstream the biogenesis of secondary vsiRNAs (Wang et al., 2011b). Actually, important antiviral roles for AGO2 have been lately described. Mutants of *ago2* were hyper-susceptible to the TCV and to the CMV (Harvey et al., 2011). During infections with these two viruses, wild plants showed an increase in AGO2 protein. In view of the interference of AGO1 activity caused by the viral suppressors of these two viruses, it is considered that AGO2 may constitute a second layer of defense against viruses. The VSR of the TCV also interact directly with AGO2 (Zhang et al., 2012). The catalytic activity of AGO2 was essential for local and systemic antiviral resistance against the TuMV (Carbonell et al., 2012). In addition, AGO2 resulted to be a key element for non-host resistance toward the *Potato virus X* (PVX; Jaubert et al., 2011). Interestingly, PVX which commonly does not infect *Arabidopsis*, was able to infect the plant in presence of the *Pepper ringspot virus* (PepRSV) that carries a viral suppressor that probably targets AGO2. In *N. benthamiana*, AGO2 plays an important antiviral role against the *Tomato bushy stunt virus* (TBSV; Scholthof et al., 2011). Besides AGO1 and AGO2, antiviral activity has been assigned also to AGO5, AGO7, and AGO4 (Qu et al., 2008; Raja et al., 2008; Takeda et al., 2008; Bhattacharjee et al., 2009; Duan et al., 2012; Hamera et al., 2012). The AGO7 protein participates in the removal of viral RNAs with less structured secondary structure (Qu et al., 2008). In the case of AGO4, viral suppressors may affect its activities to suppress its antiviral roles involving viral DNA methylation (Raja et al., 2008; Bhattacharjee et al., 2009; Duan et al., 2012; Hamera et al., 2012).

All four *Arabidopsis* DCLs perform an antiviral activity against different kind of viruses (Blevins et al., 2006; Deleris et al., 2006; Moissiard and Voinnet, 2006; Qu et al., 2008; Garcia-Ruiz et al., 2010). Their functions result essential for antiviral defense responses. DCLs may act in a hierarchical and redundant way (Blevins et al., 2006; Deleris et al., 2006; Moissiard et al., 2007). DCL4 is considered the most important DCL enzyme and the first to act against viruses in diverse interactions (Blevins et al., 2006; Bouché et al., 2006; Deleris et al., 2006; Qu et al., 2008; Garcia-Ruiz et al., 2010). In this sense, viral suppressors may affect directly or indirectly DCL4 activity to avoid this first layer of defense (Deleris et al., 2006; Qu et al., 2008). Mutants of *dcl4* altered local and systemic antiviral immunity (Garcia-Ruiz et al., 2010). DCL4 also promotes secondary siRNAs production via transitivity (Moissiard et al., 2007). DCL4-dependent virus-derived siRNAs have been described as necessary and sufficient to control a virus without its VSR (Garcia-Ruiz et al., 2010). DCL2 plays diverse roles in antiviral defense responses as well. DCL2 is the major backup during reduction of DCL4 activity by viruses. In certain viral infections and tissues, DCL2 may accomplish antiviral immunity in *dcl4* mutants (Xie et al., 2004; Bouché et al., 2006; Deleris et al., 2006; Qu et al., 2008; Garcia-Ruiz et al., 2010). Plants involving *DCL4* and *DCL2* mutants strongly overaccumulated viral RNAs. DCL2 was also required for systemic antiviral immunity in inflorescences and transitivity (Bouché et al., 2006; Deleris et al., 2006). Crucial roles in regulating viral infection against the TCV were assigned for DCL2. Higher temperature upregulates DCL2 activity and allows resistance to this virus (Zhang et al., 2012). Viral RNA levels were increased in *dcl3* mutants, albeit being considered to have a minor role in antiviral immunity against RNA viruses (Qu et al., 2008). Moderately enhanced susceptibility in *dcl3* plants challenged with the *Beet curly top virus* (BCTV) and the *Cabbage leaf curl virus* (CaLCuV) was observed (Raja et al., 2008). Mostly, DCL3 has antiviral roles against DNA viruses and presumably by inducing viral DNA methylation. Finally, as mentioned before, *DCL1* has an indirect antiviral immunity role acting as a negative regulator of DCL4 and DCL3 (Qu et al., 2008; Azevedo et al., 2010). Nevertheless, DCL1 also acts as a positive regulator in the production of virus-derived siRNAs (Blevins et al., 2006; Moissiard and Voinnet, 2006).

The DRBPs interact with DCLs to produce small RNAs. For this reason, DRBPs were analyzed to determine its possible roles in antiviral immunity (Curtin et al., 2008). The only DRB protein found to be important for antiviral defenses is DRB4. The DRB4 protein interacts with DCL4 *in vivo* to facilitate production of *trans*-acting siRNAs. In viral infections, the viral suppressor P6 of the *Cauliflower mosaic virus* (CaMV) was shown to interact with DRB4 to accomplish its replication. Furthermore, plants expressing P6 induce similar symptoms as the *drb4* mutant (Haas et al., 2008). In plants infected with the TCV, *drb4* mutants showed increased viral RNA levels (Qu et al., 2008). In addition, the role of DRB4 has been explored in *Arabidopsis* plants infected with the *Turnip yellow mosaic virus* (TYMV). *DRB4* was reported to be induced upon infection with this virus and it was observed that this protein controls viral coat protein accumulation. Besides, DRB4 was found to interact *in vitro* with a RNA translational enhancer of the TYMV, suggesting a role for DRB4 in repressing viral RNAs at a translational level (Jakubiec et al., 2012).

The small RNA methyltransferase HEN1 is another factor required for PTGS with antiviral functions. Mutant plants in *HEN1* were more susceptible to CMV and TCV infection and significantly accumulated their viral RNAs (Boutet et al., 2003; Zhang et al., 2012). Furthermore, HEN1 was found to participate in VIGS and in spreading RNA silencing in new growth through methylation of viral siRNAs. Interestingly, methylation of viral siRNAs by HEN1 was affected during the *Oilseed rape mosaic virus* (ORMV) infection, suggesting the presence of a viral suppressor with this activity in ORMV (Blevins et al., 2006). In fact, the *Zucchini yellow mosaic virus* (ZYMV) HC-Pro suppressor inhibits the activity of HEN1 *in vitro* (Jamous et al., 2011).

In *Arabidopsis*, the SILENCING DEFECTIVE 3 (*SDE3*) gene was found to disturb PTGS. SDE3 encodes a RNA helicase-like protein. Infection of *sde3* plants with CMV increased accumulation of viral RNA and severity of symptoms (Dalmay et al., 2001). The SDE3 protein has RNA helicase and AGO-binding functions that are essential for silencing a green fluorescent protein (GFP)-tagged PVX. Further, SDE3 act downstream of RDR6, and together with AGO1 and AGO2, promotes the production of secondary siRNAs (Garcia et al., 2012). In addition, the SILENCING DEFECTIVE 5 (*SDE5*) gene, a putative homolog of a human mRNA export factor, is involved in antiviral immunity. Mutants of *sde5* were hyper-susceptible to the CMV but not to the TuMV (Hernandez-Pinzon et al., 2007).

The *SGS3* has also a role in antiviral defense. The *sgs3* mutant plants exhibited enhanced susceptibility to CMV and retarded viral-induced symptoms. An hypomorphic *sgs3* mutant also overaccumulated CMV RNA (Adenot et al., 2006). Susceptibility to TuMV or to the *Turnip vein-clearing virus* (TVCV) infections was not altered in *sgs3* plants (Mourrain et al., 2000). Moreover, *sgs3* mutants increased severity of symptoms and viral DNA levels when using a DNA VIGS vector derived from the CaLCuV. Also, DNA VIGS required SGS3 (Muangsan et al., 2004).

To mount an adequate systemic antiviral defense response the primary vsiRNAs pool is amplified through the production of secondary vsiRNAs by the RDRs. For that reason, RDR1, RDR2, and RDR6/SGS2/SDE1 have been highlighted as very important and crucial factors in antiviral plant defense responses in different plant species (Mourrain et al., 2000; Yu et al., 2003; Muangsan et al., 2004; Yang et al., 2004; Qu et al., 2005, 2008; Schwach et al., 2005; Adenot et al., 2006; Diaz-Pendon et al., 2007; Donaire et al., 2008; Garcia-Ruiz et al., 2010; He et al., 2010; Wang et al., 2010, 2011b). Although in certain cases, some RDRs mutants have no effects on the susceptibility to viruses carrying VSRs (Diaz-Pendon et al., 2007; Donaire et al., 2008). RDR1 was induced upon infection with TMV in *Arabidopsis* and in tobacco. *Arabidopsis* *rdr1* plants infected with TMV showed increased levels of viral RNAs and enhanced susceptibility locally and systemically (Xie et al., 2001; Yu et al., 2003). Also, *rdr1* plants accumulated higher levels of viral RNA from a suppressor defective CMV strain (Wang et al., 2010). Tobacco plants expressing antisense RNA for *RDR1* also exhibit enhanced susceptibility to TMV and PVX (Xie et al., 2001). *RDR1* was induced in maize when infected with the *Sugarcane mosaic virus* (SCMV). Silenced *RDR1* maize plants were more susceptible to the infection of SCMV and accumulated more viral RNA (He et al., 2010). Interestingly, RDR1 was induced by SA (Xie et al., 2001; Yu et al., 2003). Double or triple mutants of RDRs, necessarily including *RDR1*, altered suppressor defective TuMV and CMV infections (Garcia-Ruiz et al., 2010; Wang et al., 2010). In the case of RDR2, it was showed that it contributes together with RDR1 and RDR6/SGS2/SDE1 to deal with TRV and suppressor defective TuMV infection (Donaire et al., 2008; Garcia-Ruiz et al., 2010). On the other hand, *rdr6/sgs2/sde1* plants exhibited enhanced susceptibility to CMV and to TCV (Mourrain et al., 2000; Qu et al., 2008). Also, this RDR was important for DNA and RNA VIGS (Muangsan et al., 2004). Hypomorphic *rdr6* mutants also over-accumulate CMV RNA (Adenot et al., 2006). *RDR6*-silenced *N. benthamiana *plants resulted to be hyper-susceptible to different viruses and temperature-dependent (Qu et al., 2005; Schwach et al., 2005). Furthermore, RDR6/SGS2/SDE1 participated in limiting systemic infection of a suppressor defective TuMV (Garcia-Ruiz et al., 2010). Likewise, RDR6 contributed together with RDR1 in reducing viral RNAs of a suppressor defective CMV strain as well (Wang et al., 2010). However, resistance to a different suppressor defective CMV strain was attributed mainly to RDR6 (Wang et al., 2011b). RDR6 also participates with RDR1 and RDR2 in antiviral defenses against TRV (Donaire et al., 2008).

## BIOGENESIS AND ROLES OF PATHOGEN-DERIVED siRNAs

Viral double-stranded RNAs are used by plants to produce vsiRNAs of ~20 to 24 nucleotides in length using several components of the silencing pathways to guide silencing of viruses genetic material. As previously mentioned, plants may also use transferred DNA from bacteria as template to produce bacteria-derived siRNAs (Dunoyer et al., 2006). Processing of viral dsRNAs by Dicer-like enzymes is not sufficient to block virus replication. Interestingly, several studies have indicated that vsiRNAs may regulate host gene expression. Although the identification of sRNAs derived from viral RNA in infected plant cells came along with the discovery of sRNAs in PTGS, a complete understanding of their biogenesis and their roles for the different type of viruses is still a challenge (Hamilton and Baulcombe, 1999).

The principal source of the viral dsRNA that serves as template to generate vsiRNAs has been extensively discussed. The viral dsRNA that is processed into vsiRNAs for viruses with a RNA genome was thought to originate mainly from the dsRNA intermediates that were needed for their genome replication; however, recent evidence suggests that highly structured single-stranded viral RNA precursors and perfect dsRNAs generated by host RDRs are important sources for vsiRNAs (Molnár et al., 2005; Donaire et al., 2008; Wang et al., 2010). In the case of viruses with a DNA genome, most of vsiRNAs are probably derived from transcriptional units, although bidirectional transcription is considered an option for vsiRNAs production (Moissiard and Voinnet, 2006). Furthermore, viral genomes have active and specific virus-derived siRNAs regions (hot-spots; Wang et al., 2010).

In accordance with the particular biochemical properties that each of the DCLs possess, vsiRNAs are generated into determined size classes. The 21-nt class of vsiRNAs is usually the most abundant in *Arabidopsis* plants infected with several (+) strand RNA viruses because of the primary role DCL4 has in antiviral defense. In absence of DCL4, the DCL2-dependent 22-nt class of vsiRNAs is the most abundant. The amount of 22-nt vsiRNAs when an active DCL4 is present constitutes only a small portion of the total vsiRNAs (Blevins et al., 2006; Bouché et al., 2006; Deleris et al., 2006; Diaz-Pendon et al., 2007; Garcia-Ruiz et al., 2010). In TCV infections carrying its DCL4-targeting suppressor, the 22-nt class size is the predominant size for vsiRNAs in the infected leaves, although 20, 21, and 22-nt vsiRNAs were found in systemic leaves. The 20-nt class size of vsiRNAs is explained as partial degradation products of DCL4 (Qu et al., 2008). DCL3 generates 24-nt vsiRNAs mainly in *dcl4/dcl2* double mutants during RNA virus infections. DCL3-dependent 24-nt vsiRNAs tend to accumulate in DNA virus infections and are actively important vsiRNAs (Blevins et al., 2006; Bouché et al., 2006; Deleris et al., 2006; Qu et al., 2008; Raja et al., 2008; Garcia-Ruiz et al., 2010). In the case of *DCL1*, as mentioned previously, it is considered to have an indirect role in the biogenesis of vsiRNAs. Whether DCL1 is able to process specific viral dsRNA structures or not has not been completely demonstrated. Low levels of 21-nt vsiRNAs have been detected in *dcl4 dcl2 dcl3* triple mutants infected with TuMV and CMV. Interestingly, 21-nt vsiRNAs from CMV were even detected in *dcl1 dcl2 dcl3 dcl4* plants, suggesting that other mechanism or RNase type III enzyme(s) could be involved (Bouché et al., 2006; Garcia-Ruiz et al., 2010).

The dsRNA-binding protein DRB4 participates in the biogenesis of the vsiRNAs derived from the *Tomato spotted wilt virus* (TSWV) and TYMV. The *drb4* plants infected with these two viruses showed reduced accumulation of 21-nt vsiRNAs (Curtin et al., 2008; Jakubiec et al., 2012). Nonetheless, the DRB4 roles during the biogenesis of vsiRNAs have been questioned because *drb4* plants infected with TCV showed only a small reduction in the level of 21-nt vsiRNAs and increased viral RNA levels (Qu et al., 2008). Similar to other endogenous sRNAs, vsiRNAs are subjected to methylation by HEN1 for their stabilization. Methylation of vsiRNAs is also important for spreading VIGS systemically (Blevins et al., 2006).

Diverse studies have proposed important roles for RDRs in vsiRNA biogenesis as well. The dsRNA generated by RDRs are significantly used to generate secondary vsiRNAs that assist antiviral defense responses and are required for systemic antiviral immunity. RDRs may use primary vsiRNAs as primers or aberrant viral RNA sequences for dsRNA synthesis. The relevance of their role in the biogenesis of vsiRNAs has been recently supported by high-throughput sequencing analyses of small RNA libraries from infected plant tissues. RDR1 and RDR6 play a direct role in the biogenesis of vsiRNAs. The amount of TMV-derived vsiRNAs was reduced in *rdr1* and *rdr6* *Arabidopsis* plants. Small RNA deep sequencing analysis showed that TMV-derived vsiRNAs mainly depended on RDR1 (Qi et al., 2009). A significant reduction in the accumulation of vsiRNAs was also observed in *rdr6* plants infected with three TCV mutants (Qu et al., 2008). The production of vsiRNAs from a CMV 2b-deletion mutant was observed to depend mainly on RDR1 activity as well (Diaz-Pendon et al., 2007). Later, analysis with a similar mutation in the 2b gene of CMV showed a cooperative role between RDR1 and RDR6 in the biogenesis of vsiRNAs (Wang et al., 2010). Recently, RDR6 was proposed to be the predominant RDR involved in silencing a different mutant of this suppressor through the production of secondary vsiRNAs (Wang et al., 2011b). The accumulation of vsiRNAs derived from TuMV was significantly reduced in *rdr1* mutant plants. RDR6 participates in the biogenesis of vsiRNAs during suppressor defective TuMV infection (Garcia-Ruiz et al., 2010). Interestingly, no changes in the accumulation of TRV vsiRNAs were detected in single *RDR* mutants (Deleris et al., 2006; Donaire et al., 2008).

The AGO proteins are also relevant members in the biogenesis of vsiRNAs. AGO proteins may cleave viral RNA templates to induce the production of secondary vsiRNAs (Wang et al., 2011b). Viral siRNAs derived from the CMV, the TYMV and the TCV viruses were found in immunoprecipitates of AGO1 (Zhang et al., 2006; Azevedo et al., 2010; Wang et al., 2011b). In addition, CMV-derived vsiRNAs were found in immunoprecipitates of AGO5 and AGO2 as well (Takeda et al., 2008; Wang et al., 2011b). Direct evidence of an AGO-induced viral RNA cleavage mediated by a specific vsiRNA or a satRNA-derived small interfering RNA (satsiRNA) has been reported (Pantaleo et al., 2007; Szittya et al., 2010; Zhu et al., 2011). However, it is uncertain if all the vsiRNAs produced during a plant–virus interaction are loaded into a particular AGO protein. Nowadays, it is known that the 5′ terminal nucleotide preference in loading a small RNA for certain AGOs is conserved also for vsiRNAs (Mi et al., 2008; Wang et al., 2011b).

It was previously mentioned that plants infected with viruses may present a wide range of disease symptoms that have been correlated with disturbances in endogenous sRNA-regulated target genes (Kasschau et al., 2003; Moissiard et al., 2007). As expected, vsiRNAs also regulate host gene targets that may have an impact in viral infections. *In silico* target-prediction analyses have proposed many host genes that could be potentially regulated by vsiRNAs. More than 100 *Arabidopsis* transcripts were found to be potentially targeted by CaMV-derived vsiRNAs. Interestingly, the mRNA At1g76950, that has a Ran GTPase binding, chromatin binding, and zinc ion binding functions, was validated as a directly vsiRNA-regulated transcript (Moissiard and Voinnet, 2006). Moreover, bioinformatic analyses revealed that many host transcripts could be also potentially targeted by TMV-derived siRNAs. For this set of putative host targets, the cleavage of two transcripts was validated by 5′ RACE assays. The two transcripts encode for a cleavage and polyadenylation specific factor (CPSF30) and an unknown protein similar to the translocon-associated protein alpha (TRAP α; Qi et al., 2009). Surprisingly, two studies reported simultaneously that the yellowing symptoms induced in *N. tabacum* by the CMV Y-satellite RNA (Y-Sat) is the consequence of the chlorophyll biosynthetic gene (*CHL1* mRNA) downregulation mediated by Y-Sat-derived siRNAs. The *CHL1* mRNA from *N. tabacum* was found to possess a 22-nt sequence site complementary to the Y-Sat. Interestingly, it was also reported that other two *Nicotiana* species that do not exhibit yellowing symptoms when infected with the CMV Y-Sat are due to a single nucleotide polymorphism presented in the *CHL1* mRNA sequence (Shimura et al., 2011; Smith et al., 2011). Finally, using sRNA and degradome data, a recent study performed in *Vitis vinifera* showed that several host transcripts were subjected to cleavage by vsiRNAs of the *Grapevine fleck virus* (GFkV) and the *Grapevine rupestris stem pitting-associated virus* (GRSPaV; Miozzi et al., 2013).

In bacteria, the widely studied pathogenic *Agrobacterium tumefaciens* is well-known for introducing a T-DNA that integrates into the genome of plants. The T-DNA encodes genes that trigger the formation of a callus that produces certain compounds called opines. These compounds are used by the bacteria as nutrients. Considering the effects of RNA silencing against foreign genetic material, it was thought that this mechanism may played an important role during this particular bacteria–plant interaction. Small RNAs from the tryptophan 2-monooxygenase (*iaaM*) oncogene and the agropine synthase (*ags*) gene were detected after 3 days of post-infiltration with *A. tumefaciens* in *N. benthamiana *leaves. Interestingly, like in several cases of viral RNA silencing, sRNAs were predominantly 21-nt long and originated from sense and antisense strands, suggesting an important role for DCL4 and RDR6 in the biogenesis of this bacteria-derived sRNAs.

## BACTERIAL AND VIRAL SUPPRESSORS OF RNA SILENCING

**FIGURE 1:**
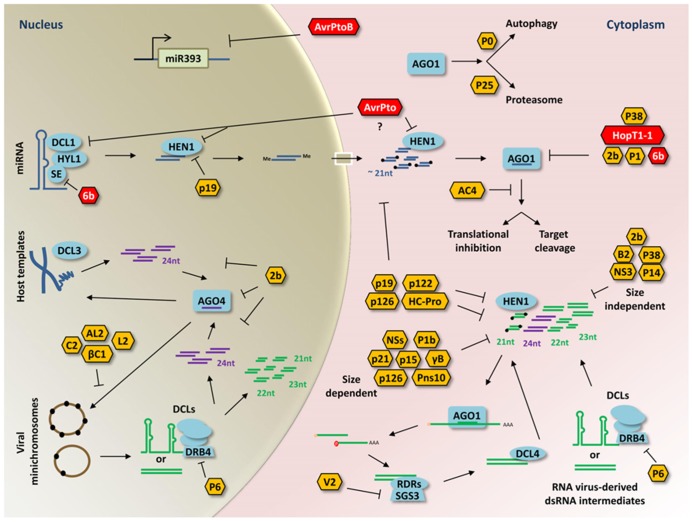
**Representative model of viral and bacterial RNA silencing suppressor functions in plants.** Double-stranded RNA intermediates are processed by DCLs to generate small RNA duplexes of different sizes. Single-stranded small RNAs are then loaded into AGO proteins to guide RNA silencing through RNA cleavage, translational repression, or DNA methylation. Most of the BSRs discussed in this review (red hexagons) target sRNA silencing pathway components involved in endogenous sRNA-regulated process such as AGO1, SE, DCL1, or HEN1. In contrast, the BSR AvrPtoB causes transcriptional repression of the At-miR393a and the At-miR393b precursors in the nucleus (gray background). The specific target of AvrPto has not been determined but it may target DCL1 or HEN1 because of its effects on miRNA accumulation (question mark). In antiviral RNA silencing, VSRs (yellow hexagons) disturb sRNA silencing pathway components as well. Several of these targeted host-factors participate in the production, stability, and function of vsiRNAs generated from DNA or RNA virus-derived dsRNA intermediates (green structures). The viral suppressor V2 interacts with the SGS3 factor and avoids sRNA amplification. The P6 protein affects production of DCL4-dependent sRNAs by interacting with DRB4. Some VSRs (P122, HC-Pro, P19, P126) have been reported to inhibit 3′ methylation of sRNAs probably by sequestering double-stranded sRNAs and/or arresting HEN1 methylation. Similar to BSRs, viral suppressors (P38, 2b, P1) interact directly with AGO1. The AC4 protein suppress PTGS by binding only single-stranded sRNAs. For DNA viruses (pararetroviruses or geminiviruses) that accumulate as minichromosomes in the nucleus (brown circles), methylation-mediated transcriptional gene silencing is affected by several viral suppressors (βC1, C2, AL2, L2, 2b). Another major strategy employed by VSRs consists on sequestering sRNAs with a specific (size-dependent) or unspecific (size-independent) nucleotide length. Furthermore, P0 and P25 promote AGO1 degradation through autophagy and proteasome-dependent degradation, respectively. Small RNA binding and disturbance or inhibition of small RNA silencing factors are indicated (perpendicular lines).

Plant–microbe interactions are sophisticated and dynamic, involving the continuous improvement of complex defense and counter-defense strategies from both sides. Several microbes introduce effector proteins into plant cells in order to suppress PTI. The contribution of sRNA-mediated silencing in PTI and ETI suggested the existence of bacterial suppressors of RNA silencing (BSRs) and VSRs. Suppressors of RNA silencing may impact small RNA silencing pathway proteins, long double-stranded RNAs, small RNAs, DNA methylation, or sRNA-derived genes to modify the biogenesis, maturation, or function of endogenous and microbe-derived small RNAs. VSRs constitute a diverse group that is widely distributed among viruses. In contrast, only few BSRs have been identified; however, VSRs and BSRs share common strategies like AGO1 disturbance (**Figure 1**).

To test if bacterial effectors evolved to suppress plants miRNAs, Navarro et al. (2008) analyzed modifications in miRNA transcription, biogenesis, or activity favored by this group of proteins. Plants treated with Pst DC3000, compared with those treated with Pst DC3000 *hrcC*^-^, presented reduced accumulation of the PAMP-responsive miRNA precursors pri-miR393a/b and pri-miR396b. The PAMP-insensitive pri-miRNAs pri-miR166a and pri-miR173 were unaltered. These results suggested that some bacteria effectors may suppress PAMP activation of *At-miR393a* and *At-miR393b* transcription. Analyzing *Arabidopsis* plants transiently transformed with different effector proteins, they found that the protein AvrPtoB, a protein with E3-ubiquitin ligase activity, function as a specific bacterial suppressor causing transcriptional repression of the At-miR393a and the At-miR393b precursors. Along with the AvrPtoB suppressor, a different effector protein called AvrPto was identified. In this case, the AvrPto suppressor caused reduction in miR393, miR171, and miR173 accumulation. In contrast to AvrPtoB, no changes in the transcription rate for these three pri-miRNAs were observed to be caused by AvrPto, indicating that AvrPto may affect miRNA biogenesis or stability. Finally, the protein HopT1-1, classified as BSRs, was observed to be involved in suppressing miRNA activity through AGO1 disruption. HopT1-1 apparently interferes with AGO1 affecting miRNA activity related to transcript degradation and translational repression (Navarro et al., 2008). Exactly how these bacterial suppressors act at the molecular level to alter miRNA transcription, biogenesis, and activity still has to be determined.

Lately, the characterization at the molecular level of the *Agrobacterium tumefaciens *6b protein from the T-DNA region of the Ti plasmid suggests that this protein may function as an RNA silencing suppressor (Wang et al., 2011a). The 6b protein interacts with AGO1 and SE *in vivo* and *in vitro*. *Arabidopsis* plants overexpressing the 6b protein presented reduced accumulation of miRNAs by targeting AGO1 and SE. Besides, plants overexpressing the 6b protein plants shared similar morphological phenotype with *ago1-27* and *se-1* mutants, and with plants overexpressing the 2b RNA silencing suppressor (Wang et al., 2011a). Although the protein 6b can interact with other proteins in the nucleus, it seems evident that this protein plays a role in RNA silencing suppression. Although it is considered that suppression of RNA silencing pathways in tumors may be a consequence of phytohormones produced as consequence of transformation, it will be interesting to determine if the protein 6b contributes to the RNA silencing suppression state observed against the T-DNA genes in tumors.

Since the identification of the first VSRs, many proteins that inhibit RNA silencing during plant–virus interactions have been identified. In many cases, these proteins have other functions besides suppressing RNA silencing and usually do not share sequence or structural similarities. Two major approaches are commonly used by VSRs to inhibit RNA silencing. One of these strategies involves direct binding of VSRs to long dsRNAs and small RNAs to avoid vsiRNAs from being stabilized or loaded into AGO proteins. VSRs like B2 (*Flock house virus*), NS3 (*Influenza A virus*), 2b (*Tomato aspermy virus* and CMV), P14 (*Pothos latent virus*), and P38 (TCV) bind size-independent dsRNAs (Jiang et al., 2012; Omarov and Scholthof, 2012). The P19 viral suppressor of tombusviruses, a widely studied protein, preferentially binds to dsRNA of 19 base pairs long; however, this suppressor could also bind sRNAs of different sizes such as DCL4-dependent 21-nt siRNAs (Scholthof, 2006). Structural analyses showed that P19 is able to measure and select small RNAs in a homodimer conformation (Silhavy et al., 2002; Vargason et al., 2003; Dunoyer et al., 2010). Likewise to P19, several suppressors including P21 (*Beet yellows virus*), P15 (*Peanut clump virus*), γB (*Barley stripe mosaic virus*), HC-Pro (*Tobacco etch virus*), P122 (TMV), NS3 (*Rice hoja blanca virus*), Pns10 (*Rice dwarf virus*), and the tospoviral NSs proteins bind mostly size-dependent double-stranded sRNAs having 2-nt 3′ overhangs (Jiang et al., 2012; Omarov and Scholthof, 2012). The P1b suppressor from the *Cucumber vein yellowing virus* (CVYV) interacts with similar affinity to double-stranded sRNAs with a phosphoryl group or a free OH at their 5′ ends and to duplexes with 2-nt 3′ overhangs or blunt-ends (Valli et al., 2011). Interestingly, the AC4 protein of the *African cassava mosaic virus* (ACMV) binds only single-stranded miRNAs and siRNAs (Chellappan et al., 2005). The second major strategy employed by VSRs to arrest the assembly of functional RISCs is carried out through the direct binding of VSRs with components of the RISC, for instance AGO1. The two viral suppressors P38 (TCV) and P1 [*Sweet potato mild mottle virus *(SPMMV)] interact with the AGO1 protein using GW/WG motifs commonly employed by some endogenous RNA silencing components to assemble functional RISCs. Point mutations in the GW residues of P38 resulted in resistant plants against the TCV infection. Interestingly, the AGO1-binding activity of the P1 protein of SPMMV allows this suppressor to inhibit mature assembled AGO1-containing RISCs and their *de novo* assembly (Azevedo et al., 2010; Giner et al., 2010). The 2b suppressor of CMV was the first suppressor identified to directly bind AGO proteins. The interaction of 2b with AGO1 blocks AGO1 cleavage activity and occurs through one surface of the Piwi-Argonaute-Zwille (PAZ)-containing module and a part of the P-element-induced wimpy testis (PIWI) domain (Zhang et al., 2006). In addition, the 2b protein interacts also with AGO4 altering the RdDM pathway. Further analysis from 2b immunoprecipitates revealed the great binding affinity this suppressor has for 24-nt repeat-associated sRNAs (Hamera et al., 2012). Surprisingly, the *Polerovirus* protein P0 that encodes an F-box protein induces AGO1 degradation. The P0 suppressor does not interact directly with AGO1, but instead interacts with the S-phase kinase-related proteins ASK1 and ASK2, two components of the SCF (SKP1/Cullin1/F-box/RBX) E3 ubiquitin ligase complex. For this reason, it was thought that P0 promoted AGO1 ubiquitination and degradation via the ubiquitin proteasome system (UPS); however, a recent report showed that P0-induced degradation of AGO1 occurs through the autophagy pathway (Pazhouhandeh et al., 2006; Baumberger et al., 2007; Derrien et al., 2012). Another viral suppressor that triggers AGO1 degradation is the P25 protein encoded by the PVX. In contrast with P0, this suppressor indeed promotes proteasome-dependent degradation of AGO1. Also, P25 may interact with AGO2, AGO3, and AGO4 (Chiu et al., 2010).

Specific viral suppressors that employ different and particular strategies to avoid RNA silencing have been identified as well. For example, the V2 protein of TYLCV is a RNA silencing suppressor that interacts directly with SGS3 and binds dsRNA. Disruption of V2–SGS3 interaction with a point mutation version of V2 stops RNA silencing suppression. These results suggested that V2 affects the interaction of SGS3–RDR6 to avoid small RNA amplification (Glick et al., 2008). Notably, the dsRNA-specific class 1 RNA endoribonuclease III (RNase3) of the *Sweet potato chlorotic stunt virus* (SPCSV) acts as a silencing suppressor using a particular approach that involves its endonuclease activity. This RNase was shown to cleave small RNAs of 21, 22, and 24-nt long into unfunctional small RNAs of 14 base pair size (Cuellar et al., 2009). The viral translational *trans*-activator protein P6 of the CaMV also abolishes RNA silencing. The P6 protein interacts with the dsRNA-binding protein DRB4, which interacts with DCL4 to produce 21-nt siRNAs. This means that P6 suppress DCL4-dependent vsiRNAs production (Haas et al., 2008). Other VSRs including P122, HC-Pro, P19, P126 have been reported to inhibit 3′ methylation of small RNA duplexes probably by sequestering double-stranded small RNAs and blocking HEN1 methylation (Jiang et al., 2012; Omarov and Scholthof, 2012). Furthermore, several VSRs from DNA viruses have been shown to suppress or alter the PTGS mechanism that regulates DNA methylation and histone adjustments. The AL2 protein of *Tomato golden mosaic virus* (TGMV) and the L2 protein of BCTV suppress RNA silencing by inhibiting the adenosine kinase (ADK) activity, which plays relevant roles in adenosine salvage and methyl cycle maintenance. Inactivation of ADK resulted in suppression of RNA silencing as occurred with the incorporation of the two geminivirus proteins. These two proteins can reverse TGS of a GFP transgene introduced in *N. benthamiana*. In addition, the AL2 and L2 proteins were found to cause ectopic expression of an endogenous loci silenced by methylation and a global reduction in cytosine methylation (Wang et al., 2005; Buchmann et al., 2009). Another viral suppressor that affects methylation modifications is the C2 protein of the *Beet severe curly top virus* (BSCTV). The C2 protein interacts with the *S*-adenosyl-methionine decarboxylase 1 (SAMDC1) attenuating its proteasome-dependent degradation. The SAMDC1 protein participates in the polyamine biosynthesis, but is also important for SAM/dcSAM balance and transmethylation (Zhang et al., 2011c). The betasatellite of the *Tomato yellow leaf curl China virus* (TYLCCNB) encodes a protein called βC1 that acts as a suppressor of methylation-mediated TGS. The βC1 protein interacts with the *S*-adenosylhomocysteine hydrolase (SAHH) enzyme that is involved in the methyl cycle and therefore plays a role in TGS. The expression of the βC1 protein decreases cytosine methylation of the viral and host genomes. Also, this protein was shown to reverse TGS applied to a transgene and an endogenous locus (Yang et al., 2011). As previously mentioned, many VSRs with small RNA-binding activities and/or capable of interacting with important sRNA silencing pathway components, may modify the levels of endogenous sRNAs like miRNAs and ta-siRNAs (Shimura and Pantaleo, 2011).

## CONCLUDING REMARKS

The small RNA-mediated plant defense responses have emerged as relevant components of the innate immune system. Increasing evidence has highlighted the warfare that takes place between plants and microbes around the RNA silencing system. In this review, the recent findings, similarities and differences related to the RNA-mediated arms race between plants and two important group of microbes such as bacteria and viruses were discussed. In general, evident biological differences between these two groups of microbes are reflected in big differences regarding small RNA-mediated antiviral and antibacterial immunity; however, there are also specific similarities in plants defense responses through RNA silencing against bacteria and viruses. Likewise, similar strategies have been identified related to the microbes counter defense responses against RNA silencing. Bacteria-responsive miRNAs with potential roles in regulating bacterial immunity have been reported for several plant–bacteria strains interactions, flg22 treatment and even during symbiotic nitrogen fixing bacteria inoculation and nodule development (Subramanian et al., 2008; Lelandais-Brière et al., 2009; Wang et al., 2009; De Luis et al., 2012; Reynoso et al., 2012). Most of these miRNAs are involved in hormone signaling pathways and ETI. In viral infections, few miRNAs have been identified to play a direct role in antiviral immunity because viruses usually affect the RNA silencing system to circumvent this kind of plant defense response. Additionally, viral infections induce expression of novel phased miRNAs from conserved miRNA precursors (Du et al., 2011). Although, sRNA-regulated genes involved in ETI during bacterial infections are well-documented, it still remains to be evaluated to what extent host small RNAs participate in antiviral immunity. In this regard, several miRNAs have been proposed to be good candidates to directly act as vsiRNAs regulating viral RNAs (Perez-Quintero et al., 2010). Comparing the antiviral and the antibacterial roles of the small RNA biogenesis factors may shed light on the complex modes of regulation these proteins have to confer plants disease resistance. The study of VSRs and BSRs along with their targets may help to decipher redundancy in the activity of several RNA silencing components during plant–microbe interactions. Further studies related to this growing field will define more precisely the global small RNA-mediated plant defense responses induced by bacteria and viruses. We expect that understanding small RNA responses to viral and bacterial infections will provide novel means to generate disease-resistant plants.

## Conflict of Interest Statement

The authors declare that the research was conducted in the absence of any commercial or financial relationships that could be construed as a potential conflict of interest.
